# Identifying cut‐off scores for interpretation of the Heart Failure Impact Questionnaire

**DOI:** 10.1002/nop2.168

**Published:** 2018-07-16

**Authors:** Tsui‐Wen Hsu, Hui‐Chin Chang, Chi‐Hung Huang, Ming‐Chih Chou, You‐Tsz Yu, Long‐Yau Lin

**Affiliations:** ^1^ Institute of Medicine Chung Shan Medical University Taichung Taiwan; ^2^ Department of Nursing Cathay General Hospital Taipei Taiwan; ^3^ School of Public Health Chung Shan Medical University Taichung Taiwan; ^4^ Chung Shan Medical University Hospital Taichung Taiwan; ^5^ Department of Internal Medicine Cathay General Hospital Taipei Taiwan; ^6^ School of Medicine Chung Shan Medical University Taichung Taiwan; ^7^ Department of Surgery Chung Shan Medical University Hospital Taichung Taiwan; ^8^ Department of Family and Community Medicine Chung Shan Medical University Hospital Taichung Taiwan; ^9^ Evidence‐based Medicine Center Chung Shan Medical University Hospital Taichung Taiwan; ^10^ Department of Obstetrics and Gynecology Chung Shan Medical University Hospital Taichung Taiwan

**Keywords:** 36‐Item Short‐Form Health Survey, heart failure, Minnesota Living with Heart Failure Questionnaire, nurses, nursing, quality of life

## Abstract

**Aims:**

Heart failure (HF) influences health‐related quality of life. However, the factors that contribute to health‐related quality of life remain unclear in Taiwan. We aim to identify the factors influencing health‐related quality of life in HF patients.

**Methods:**

Hospitalized HF (*N* = 225) patients were included from April 2011 to April 2014. Health‐related quality of life was assessed by using the 36‐Item Short‐Form Health Survey (SF‐36) and the Minnesota Living with Heart Failure Questionnaire. A new cut‐off was conducted based on the combination of SF‐36 and Minnesota Living with Heart Failure questionnaire.

**Results:**

There were significant differences between good and poor quality groups on age, gender, education levels, occupational classification caregiver, New York Heart Association classes, and the numbers of comorbidities. The logistic regression analysis showed that the number of comorbidities was more than three and New York Heart Association class IV were significantly associated with health‐related quality of life.

## 1 INTRODUCTION

Heart failure (HF) is an eventual outcome of the complex clinical problems caused by various underlying conditions; due to various physical and psychological symptoms of the HF, it is a major global health issue (Norton, Georgiopoulou, Kalogeropoulos, & Butler, 2011; Soriano et al., 2010). HF also presents a socioeconomic burden, which influences personal ailment tolerance and family care devolution, and significantly contributes to leading cause of all hospitalizations and readmissions in older people (Hwang, Liao, & Huang, 2014; Jeon, Kraus, Jowsey, & Glasgow, 2010).

Traditional guidance on HF treatments are to improve prognosis, symptom control, and relieving uncomfortability. The ultimate goal predictor and complimentary end point is to evaluate the use of clinical trails in HF therapy and to induce remissions in HF progress. (Guyatt, 1993; Harrison et al., 2002; Janz et al., 2001; Lee, Yu, Woo, & Thompson, 2005; Siegrist & Junge, 1990; Stull, Clough, & Van Dussen, 2001; Wenger, 1989). The measurements of quality of life (QOL) in most of the studies have been performed based on the hospital setting and discharge follow‐up (McMurray et al., 2012; Naveiro‐Rilo et al., 2010; Santos, Plewka, & Brofman, 2009).

Health‐related quality of life (HRQOL) is defined as a multidimensional concept that has an impact on the daily lives activity performances of patients with chronic diseases including patients’ functional capabilities, symptoms, and psychosocial perceptions on overall well‐being (Heo, Doering, Widener, & Moser, 2008; Jaarsma et al., 2000; Yu, Lee, & Woo, 2004).

In HF patients, HF disease scenario is chronic and prognostic situations. Therefore, while evaluating QOL, there are two important types of QOL score system questionnaires, one is the Minnesota Living with Heart Failure questionnaire (MLFHQ) and other is the 36‐Item Short‐Form Health Survey (SF‐36). Among the two scoring system, the most frequently used for both generic and disease‐specific measures. The MLHFQ subscales screening for physical dimension and emotional dimension to give more specific information about a special group and reveals more sensitive results (Behlouli et al., 2009; Bilbao, Escobar, García‐Perez, Navarro, & Quirós, 2016). The other instrument is the SF‐36, which is the generic measure of QOL and gives validated, reliable and multidimensional results. The SF‐36 consists of eight domains; the scores of these subscales can be combined to create two higher order summary scores: the physical component summary (PCS) and mental component summary (MCS). These results can be compared with those of a general population to give a more information on general health status (Huber, Oldridge, & Hofer, 2016; Ware & Sherbourne, 1992; Wylie, Beckmann, Granger, & Tashjian, 2014). The weighing of other QOL domains for combining two scores include psychosomatic symptomatology and emotional interference.

Correlations between all scores were calculated in previous studies. Numerical values allow evaluation of patient change. The classification of QOL scores may be helpful to take a multifaceted decision‐making for the implementation of treatment. However, the question is: which QOL measuring instrument is more accurate? Is it possible to define a new cut‐off point using the combination of MLHFQ and SF‐36?

To solve this question, in this study, we aimed to: (1) assess the combination of SF‐36 and MLHFQ, and describe the sensitivity and specificity of cut‐off scores in screening for HF; and (2) study the diagnostic properties and diagnostic values of SF‐36 and MLHFQ in predicting HF patients QOL score.

## 2 THE STUDY

### 2.1 Design

The present cross‐sectional study was collected data by face‐to‐face interviews with the participants at clinical sites. In total, 225 HF patients were enrolled from Cathay General Hospital in Northern Taiwan. The recruitment period was from April 23, 2011‐April 30, 2014.

Inclusion criteria were the following: (1) a diagnosis of HF (both systolic and diastolic failure) by a physician and assessed based on New York Heart Association (NYHA) class II–IV heart disease for at least 3 months; (2) abnormalities of focal ventricular motion, abnormal left ventricular end‐diastolic dimension, systolic dysfunction, or valves abnormalities detected by echocardiography; (3) left‐ventricular ejection fraction (LVEF) <40%; (4) hospitalized at least twice due to HF; and (5) ability to engage in conscious and coherent verbal communication with the interviewer. Patients who: (1) were diagnosed with mental disorders; (2) were bedridden for >3 months and unable to ambulate; (3) had severe visual or hearing impairment; and (4) refused to participate were excluded from this study.

### 2.2 Method

The MLHFQ is specifically designed for evaluating HF patients’ to understand their disease status as well as their QOL within 1 month after the completion of primary treatment (Heo, Moser, Riegel, Hall, & Christman, 2005). It is composed of 21 items which cover HF‐related physical, psychological and social impairments. The questions are calculated on a Likert‐type scale that ranges from 0 to 5 and can be summarized to a total score of highest 105. Lower scores indicate better HRQOL. The content validity index was 0.98. The construct validity was supported by exploratory factor analysis in a Chinese version. The instrument demonstrated high internal consistency (Cronbach's α of 0.95 for the scale, and 0.93 and 0.95 for the physical dimension and emotional dimension subscales, respectively) (Bennett et al., 2003; Ho, Clochesy, Madigan, & Liu, 2007; Middel et al., 2001; Rector & Cohn, 1992). The MLHFQ scores <24, 24–45, and >45 point were classified as good, moderate, and poor, respectively (Behlouli et al., 2009).

The SF‐36 measures perceived health status in eight dimensions: physical function, role limitations due to physical problems, body pain, general health, vitality, social function, role limitations due to emotional problems, and general mental health. The scores were summarized into two component summary scores of physical and mental health. Scores range from 0 (worst)‐100 (best). The Cronbach's α of internal consistency reliability were 0.72 and 0.88, respectively. The 2‐week test–retest reliability coefficients were 0.66 and 0.94, respectively. The cut‐off point of SF‐36 score ≥60 was suggested as good physical function (Bieleman et al., 2009; Garratt, Ruta, Abdalla, & Russell, 1994; Ware & Sherbourne, 1992). Thus, each patient's HRQOL was measured by using a generic instrument (SF‐36) and an HF‐specific instrument (MLHFQ).

### 2.3 The combination of SF‐36 and MLHFQ

**Figure 1:**
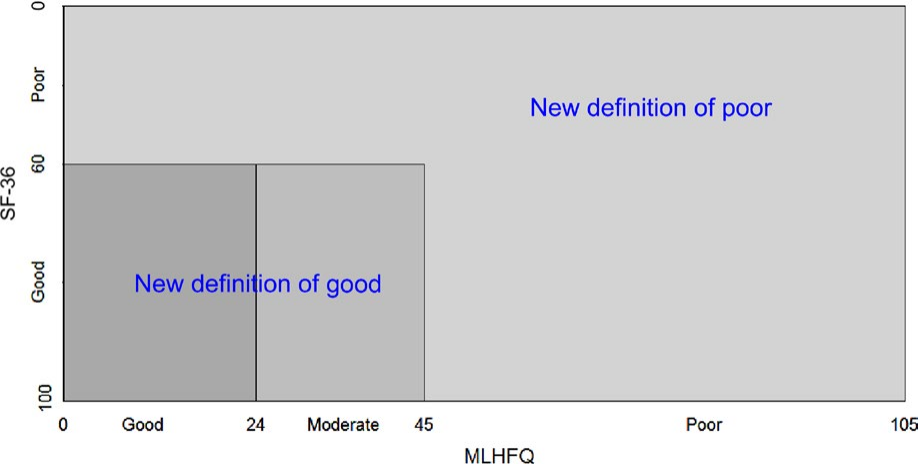
Matrix on combination measurement of QOL in SF‐36 and MLHFQ

**Figure 2:**
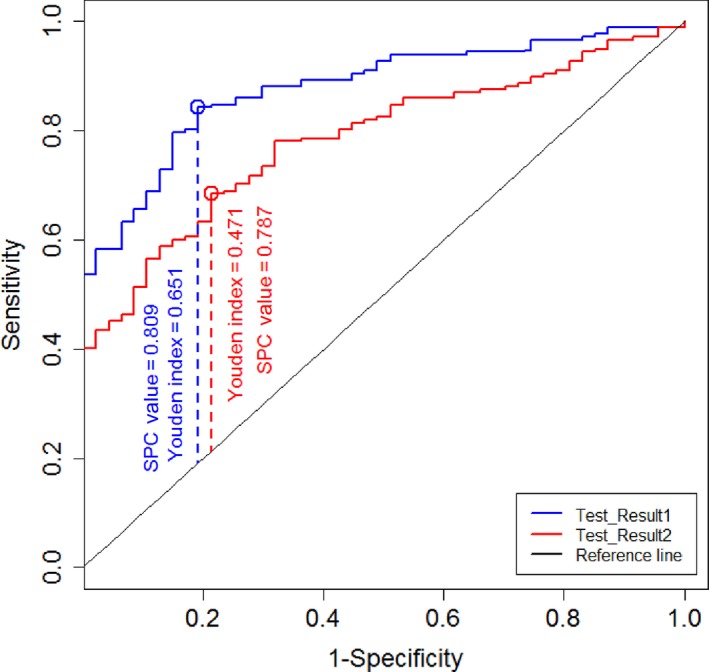
The area under the ROC curve of (1) 0.2 × SF‐36 score + 0.8 × MLHFQ score; and (2) 0.3 × SF‐36 score + 0.7 × MLHFQ score for the new definition of quality of life

To construct the new cut‐off, the new definition for good HRQOL was as follows: (1) MLHFQ score <24; (2) patients with MLHFQ score <45 and SF‐36 score ≥60. The new definition for poor HRQOL was as follows: (1) MLHFQ score ≥45; (2) MLHFQ score ≥24 and SF‐36 score <60 (Figure 1). The weighted HRQOL index was verified by the area under the receiver operating characteristic (ROC) curve. Two weighted total HRQOL scores were as follows: (1) 20% of SF‐36 score and 80% of MLHFQ score; and (2) 30% of SF‐36 score and 70% of MLHFQ score (Figure 2).

### 2.4 Analysis

Descriptive statistics were expressed as the mean ± standard deviation (*SD*) for continuous variables, and as the frequencies and percentages for categorical variables. Chi‐square test and Student's *t* test were used to test the differences between the two groups. The SF‐36 and MLHFQ were analysed for the continuous sociodemographic and clinical characteristics by Pearson's correlation. Logistic regression analysis was used to investigate the associations between HRQOL measures and clinical characteristics. A two‐tailed test with α < 0.05 was considered to be statistically significant. All analyses were conducted by using SPSS statistical software (version 18.0; SPSS Inc., Chicago, IL, USA).

### 2.5 Ethics

This study was approved by the Institutional Review Board of Cathay General Hospital. All participants agreed to participate in the research and signed an informed consent form.

## 3 RESULTS

### 3.1 Patient characteristics

Baseline characteristics and clinical data in all participants with HF were collected. Most of the patients were retired or unemployed; the mean age was 70.88 years and 65% were male. Most the patients (70.18%) were in NYHA functional class III or IV. Only 56% of participates were married, but most (85%) of them lived with their family and had a low educational level. The mean body mass index (BMI) was 25.60 kg/m^2^ (*SD* 4.11) and all patients had at least two comorbid illnesses. Patients with MLHFQ scores 24–45 as moderate were younger, had higher education, and low NYHA class as compared with those with MLHFQ >45 (Table 1). Similarly, patients with SF‐36 scores ≥60 were younger, had higher education, higher probability of not having caregivers, lower NYHA class, and fewer comorbidities compared with those with SF‐36 <60 (Table 2).

**Table 1:** Characteristics of patients with MLHFQ score <24, 24–45, and ≥45

Variable	Good (*N* = 17)	Moderate (*N* = 72)	Poor (*N* = 136)	*p* Value
	Mean ± *SD* or *N* (%)	Mean ± *SD* or *N* (%)	Mean ± *SD* or *N* (%)	
Age (years)	70.88 ± 88.24	65.62 ± 15.53	72.55 ± 14.83	<0.01
Sex				
Male	12 (70.59%)	42 (58.33%)	79 (58.09%)	0.61
Female	5 (29.41%)	30 (41.67%)	57 (41.61%)	
Marital status				
Married	11 (64.71%)	40 (55.56%)	75 (55.15%)	0.75
Other	6 (35.29%)	32 (44.44%)	61 (44.85%)	
Educational level				
Elementary school	7 (41.18%)	23 (31.94%)	83 (61.03%)	<0.01
Junior and senior high school graduates	6 (35.29%)	40 (55.56%)	38 (27.94%)	
University graduate or higher degree	4 (23.53%)	9 (12.50%)	15 (11.03%)	
Occupational classification				
Government employee	2 (11.76%)	8 (11.11%)	5 (3.68%)	0.03
Skilled workers	6 (35.29%)	21 (29.17%)	25 (18.38%)	
Other	9 (52.94%)	43 (59.72%)	106 (77.94%)	
Living arrangements				
Alone	2 (11.76%)	12 (16.67%)	13 (9.56%)	0.32
With others	15 (88.24%)	60 (83.33%)	123 (90.44%)	
Caregivers				
Self	3 (17.65%)	33 (45.83%)	34 (25.00%)	<0.01
Spouse	10 (58.82%)	22 (30.56%)	37 (27.21%)	
Child	4 (23.53%)	13 (18.06%)	35 (25.74%)	
Others^a^	0	4 (5.56%)	30 (22.06%)	
NYHA classification				
Class II	9 (52.94%)	36 (50.00%)	43 (31.62%)	0.06
Class III	6 (35.29%)	19 (26.39%)	54 (39.71%)	
Class IV	2 (11.76%)	17 (23.61%)	39 (28.68%)	
BMI (kg/m^2^)	25.57 ± 2.42	25.59 ± 4.98	25.14 ± 5.61	0.82
Number of comorbidities	1.76 ± 0.66	2.64 ± 1.42	4.18 ± 2.30	<0.01
Hospitalization frequency	2 (0–6)	1 (0–7)	0 (0–11)	<0.01

Note

BMI: body mass index; NYHA: New York Heart Association; *SD*: standard deviation.

a Others represent spouse or equivalent.

**Table 2:** Characteristics of patients with SF‐36 score ≥60 and patients with SF‐36 score <60

Characteristic	Good (*N* = 62)	Poor (*N* = 163)	*p* Value
	Mean ± *SD* or *N* (%)	Mean ± *SD* or *N* (%)	
Age (years)	65.37 ± 15.19	72.04 ± 14.66	<0.01
Sex			
Male	41 (66.13)	92 (56.44)	0.18
Female	21 (33.87)	71 (43.56)	
Marital status			
Married	38 (61.29)	88 (53.99)	0.32
Other	24 (38.71)	75 (46.01)	
Educational level			
Elementary school	23 (37.10)	90 (55.21)	0.04
Junior and senior high school graduates	31 (50.00)	53 (32.52)	
University graduate and higher degree	8 (12.90)	20 (12.27)	
Occupational classification			
Government employee	5 (8.06)	10 (6.13)	0.09
Skilled workers	20 (32.26)	32 (19.63)	
Other	37 (59.68)	121 (74.23)	
Living arrangements			
Alone	7 (11.29)	20 (12.27)	0.84
With others	55 (88.71)	143 (87.73)	
Caregivers			
Self	28 (45.16)	42 (25.77)	<0.01
Spouse	22 (35.48)	47 (28.83)	
Child	9 (14.52)	43 (26.38)	
Others^a^	3 (4.84)	31 (19.02)	
NYHA classification			
Class II	42 (67.74)	46 (28.22)	<0.01
Class III	14 (22.58)	65 (39.88)	
Class IV	6 (9.68)	52 (31.90)	
BMI (kg/m^2^)	25.60 ± 4.11	25.21 ± 5.60	0.61
Number of comorbidities	2.65 ± 1.64	3.83 ± 2.23	<0.01
Hospitalization frequency	1 (0–6)	0 (0–11)	

Note

BMI: body mass index; NYHA: New York Heart Association; *SD*: standard deviation.

a Others represent spouse or equivalent.

### 3.2 MLHFQ and SF‐36

The total score of MLHFQ had positive association with old age, more severe NYHA classification, higher number of comorbidities, while it had negative association with higher educational level and higher hospitalization frequency. In addition, there were positive association between SF‐36 scores and higher BMI. However, SF‐36 scores had negative association with higher age, more severe NYHA class, and higher number of comorbidities (Table 3).

**Table 3:** MLHFQ/SF‐36 total score and explanatory characteristics

Explanatory variables	MLHFQ total score		SF‐36 total score	
	r*	*p* Value	r*	*p* Value
Age	0.19	0.00	−0.225	<0.01
Educational level	−0.13	0.05	0.119	0.08
Living arrangements	0.06	0.35	0.011	0.87
BMI	−0.01	0.84	0.144	0.03
NYHA classification	0.24	<0.00	−0.431	<0.01
Number of comorbidities	0.43	<0.00	−0.375	<0.01
Hospitalization frequency	−0.15	0.03	0.014	0.84

Note

BMI: body mass index; NYHA: New York Heart Association; *SD*: standard deviation.

Others represent spouse or equivalent.

In relation to discriminative validity, the MLHFQ total score and SF‐36 dimensions were able to distinguish characteristics of patients with new definition of good QOL (*N* = 47) and that with new definition of poor QOL (*N* = 178). Only living arrangements, married status, and BMI were not statistically significant (Table 4).

**Table 4:** Characteristics of patients with new definition of good QOL and that with new definition of poor QOL

Characteristic	Good (*N* = 47)	Poor (*N* = 178)	*p* Value
	Mean ± *SD* or *N* (%)	Mean ± *SD* or *N* (%)	
Age (years)	66.27 ± 14.33	71.24 ± 15.13	0.04
Sex			
Male	35 (74.47)	98 (55.06)	0.02
Female	12 (25.53)	80 (44.94)	
Marital status			
Married	30 (63.83)	96 (53.93)	0.22
Other	17 (36.17)	82 (46.07)	
Educational level			
Elementary school	14 (29.79)	99 (55.62)	0.01
Junior and senior high school graduates	24 (51.06)	60 (33.71)	
University graduate and higher degree	9 (19.15)	19 (10.67)	
Occupational classification			
Government officials	5 (10.64)	10 (5.62)	0.04
Skilled workers	16 (34.04)	36 (20.22)	
Other	26 (55.32)	132 (74.16)	
Living arrangements			
Alone	6 (12.77)	21 (11.80)	0.86
With others	41 (87.23)	157 (88.20)	
Caregivers			
Self	18 (38.30)	52 (29.21)	0.01
Spouse	20 (42.55)	49 (27.53)	
Child	8 (17.02)	44 (24.72)	
Others^a^	1 (2.13)	33 (18.54)	
NYHA classification			
Class II	29 (61.70)	59 (33.15)	<0.01
Class III	12 (25.53)	67 (37.64)	
Class IV	6 (12.77)	52 (29.21)	
BMI (kg/m^2^)	25.92 ± 4.05	25.16 ± 5.50	0.29
Comorbidities	2.21 ± 1.18	3.85 ± 2.22	<0.01
Hypertension	32 (68.09)	113 (63.48)	0.56
Coronary artery disease	1 (2.13)	28 (15.73)	0.01
Diabetes	16 (34.04)	70 (39.33)	0.51
Atrial fibrillation	1 (2.13)	18 (10.11)	0.08
Benign prostatic hypertrophy	2 (4.26)	20 (11.24)	0.15
Hyperlipidemia	1 (2.13)	15 (8.43)	0.14
Gout	0	12 (6.74)	0.07
Glaucoma	1 (2.13)	10 (5.62)	0.32
Sick sinus syndrome	0	6 (3.37)	0.20
Hospitalization frequency	2 (0–6)	0 (0–11)	

Note

BMI: body mass index; NYHA: New York Heart Association; *SD*: standard deviation.

a Others represent spouse or equivalent.

The logistic regression analysis for the new cut‐off of HRQOL showed a negative associations with ≥3 comorbidities (odds ratio [OR] = 4.86, 95% confidence interval [CI] 2.20–10.75) and NYHA class IV heart disease (OR = 3.79, 95% CI 1.38–10.45) (Table 5).

**Table 5:** Risk factors for the quality of life in heart failure patients: the result of logistic regression using new definition of quality of life index

	Odds ratio	95% Wald CI
Model (0: good, 1: poor)		
Age	0.97	0.93–1.00
Man (vs. women)	0.56	0.26–1.36
BMI	0.99	0.92–1.06
Number of comorbidities ≥3 (vs. <3)	4.86	2.20–10.77^a^
NYHA class IV (vs. II and III)	3.79	1.36–10.45^a^
Educational level	0.73	0.41–1.28
Married (vs. others)	0.89	0.43–1.89
Occupational classification	1.07	0.56–2.01
Caregiver	1.62	1.01–2.61^a^

Note

CI: confidence interval; MLHFQ: Minnesota Living with Heart Failure Questionnaire; NYHA: New York Heart Association.

a Statistically significant.

## 4 DISCUSSION

The results from our study showed that by using the combination of SF‐36 and MLHFQ to divide HF patients into good HRQOL and poor HRQOL groups, the number of factors associated with HRQOL was more than individual questionnaire, suggested to be a new classification for HRQOL. In addition, comorbidities were the most powerful predictors of HRQOL and NYHA functional class was strongly associated with clinical outcomes as well.

It is known that the NYHA functional classification is characterized by the severity of the HF symptoms and reflect the severity of HF, higher NYHA class may also affect QOL, reflecting the impact daily activity, limited functional abilities, and social relationship resulting from their condition (Ahmed, Aronow, & Fleg, 2006; Holland, Rechel, Stepien, Harvey, & Brooksby, 2010; Lewis et al., 2007). Therefore, researchers should assess patients’ physical and psychological symptoms and provide interventions based on symptom assessment to improve QOL.

Patients with advanced HF had more comorbidities, more clinical signs and symptoms that negatively affected HRQOL. Previous studies found that HF often coexists with diabetes, kidney, and anaemia related disease significantly related to HF‐related hospitalization and all‐cause mortality in HF patients (McMurray et al., 2012; van Deursen et al., 2014). In our result, 145 out of 225 patients had hypertension, 86 patients had diabetes, and 29 patients had coronary artery disease (Table 4). These diseases are highly related with HF. To our knowledge, this is the first study to describe and compare two instruments measure to predict HF patients’ HRQOL to explore risk factors. From this analysis, newly identified questionnaires cut‐off scores were assigned to the “good” and “poor” QOL category. It is important that the range of scores questionnaires overlapped, and therefore, they were merged to form a new score system. From this analysis, we believe this study has direct clinical importance. The data form SF‐36 in our participants were able to distinguish different levels of HF, mostly HF patients with high score by 72% (163) and form MLHFQ high score by 60% (136), we considering the HF patients in the “poor” QOL category overall dimension. However, there was a significant change between the two clusters. The decision was focus to on the assessment tools necessary to give more information on the evaluation of patients with HF; this was based on the need to identify the disease influences on the patients’ QOL, and more clearly, on different factors that may have an impact on QOL. Furthermore, the studies would follow‐up the relationship between these two tools in evaluating the QOL in HF patients and studies should focus on the validation of the combination of these two questionnaires by conducting a study which has larger sample size.

### 4.1 Limitations

The following are the limitations of this study. First, a convenience sample of patients was enrolled during hospitalization at a single medical centre. Thus, our sample might not represent all the HF population in Taiwan. However, our sample size was relatively larger than those reported in previous studies. Second, the cross‐sectional design precluded a cause–effect analysis and future studies with longitudinal design are accordingly warranted. Finally, although we included a variety of clinical characteristics, other factors, such as exercise, body weight, daily activities socioeconomic factors, family support system and 6‐min walk test, that may also influence HRQOL were not included. Future studies should consider them to explore more ADL in HF patients.

## 5 CONCLUSION

Several factors were associated with HF disease. HF is a complex entity and greatly impaired in HF patients according to the MLHFQ and SF‐36 scales. Healthcare systems should understand the multifactorial of HF patients of progressive to achieve more effective management. By screening for patients, we achieve early detection and disclose the risk factors to help HF patients to improve outcome and delay the process to irreversible HF and mortality. Moreover, assessing HF disease severity and HRQOL is also important to monitor patient care. HF is a multifactorial problem and its management requires a combination of intervention strategies.

## CONFLICT OF INTEREST

No conflict of interest has been declared by the authors.

## AUTHOR CONTRIBUTIONS

All authors have agreed on the final version and meet at least one of the following criteria [recommended by the ICMJE (https://www.icmje.org/recommendations/)]:

- substantial contributions to conception and design, acquisition of data or analysis and interpretation of data;
- drafting the article or revising it critically for important intellectual content.
